# Assessment of the stability of antimicrobials and resistance genes during short- and long-term storage condition: accounting for uncertainties in bioanalytical workflows

**DOI:** 10.1007/s00216-023-04874-6

**Published:** 2023-08-01

**Authors:** Like Xu, Barbara Kasprzyk-Hordern

**Affiliations:** 1 Department of Chemistry, University of Bath, Claverton Down, Bath, BA2 7AY UK; 2 Institute for Sustainability, University of Bath, Claverton Down, Bath, BA2 7AY UK; 3 Water and Innovation Research Centre, University of Bath, Claverton Down, Bath, BA2 7AY UK

**Keywords:** Stability, Freezing storage, Antimicrobials, Antibiotic resistance genes

## Abstract

Unravelling complexities in antimicrobial agent–microbe interactions in the context of antimicrobial resistance (AMR) requires robust analytical workflows accounting for all uncertainties. Temporal storage of wastewater samples under refrigerated or frozen conditions prior to chemical and biological analysis is widely used to facilitate laboratory routine but may affect stability of analytes over time. Yet, little knowledge exists regarding stability of biological and chemical determinants in environmental samples, which hampers validity of research outputs. This study examines, for the first time, the stability of 32 antimicrobials (AAs) including commonly used classes of antibiotics and their representative metabolites and variation of 5 antibiotic resistance genes (ARGs) (*ermB*, *sul1*, *tetW*, *blaCTX-M*, *qnrS*), as well as *intI1* and *16S rRNA* genes in a reference wastewater sample stored under freezing condition for up to 1 year. Ultra-performance liquid chromatography coupled with tandem mass spectrometry (UPLC-MS/MS) and quantitative PCR (qPCR) techniques were adopted to measure concentration of AAs and ARGs, respectively. Results suggested that parent compounds are less affected by freezing storage compared to the metabolites. β-Lactams, clindamycin, and N-desmethyl clindamycin are the most-affected compounds which were poorly recovered (34–67%) from the starting concentration. By contrast, sulfonamides, macrolides, quinolones, and azoles are generally stable under freezing condition. No consistent differences were observed in gene copies between fresh and frozen samples, and *ermB* and *tetW* showed the highest variabilities at 30% under freezing condition. Overall, this study adds to the current knowledge on environmental AMR monitoring and emphasises the need for standardised protocols for AMR monitoring in the wastewater samples.

**Graphical Abstract:**

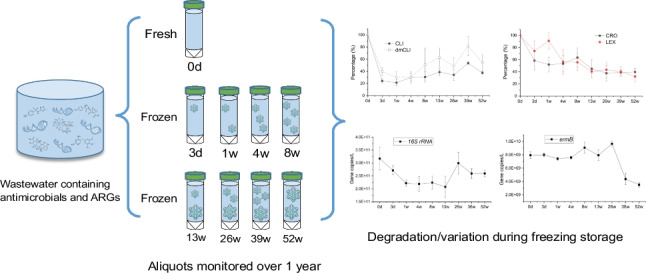

**Supplementary Information:**

The online version contains supplementary material available at 10.1007/s00216-023-04874-6.

## Introduction

The World Health Organization (WHO) has recognised antimicrobial resistance (AMR) as one of the most significant global public health threats facing humanity, and it requires a collaborative action plan to address the spread of AMR globally. However, the lack of surveillance data in many regions is one of the major challenges that needs to be resolved [1]. Wastewater-based epidemiology (WBE), as a promising approach for the environmental AMR surveillance, could track the status of AMR and identify epidemiological links between humans, animals, and the environment within the “One Health” domain. WBE utilises the analysis of wastewater to provide insight into the health of communities and has been so far applied to estimate alcohol and cocaine co-consumption [2], public exposure to illicit drug [3], tobacco [4], antibiotics and corresponding resistance genes [5], and stress biomarkers [6].

In the WBE domain, as well as the wider environmental AMR surveillance, there are a few published researches on both antibiotics and the corresponding antibiotic resistance gene (ARG) indicators. The presence of quinolones, macrolides, sulfamethoxazole, and chloramphenicol, as well as the corresponding ARGs in European wastewaters and a river catchment area in the Southwest of England, has been investigated in previous studies [5, 7, 8]. Wastewater-based surveillance has been recently adopted in a global metagenomics-based study identifying the geographic “hot spots” for ARGs in 6 countries [9]. Core antibiotic resistome has been successfully identified in different urban communities in Singapore via sewage metagenome surveillance [10]. With the increasing national and international collaborations on AMR research, it is usually common practice sending wastewater samples from site to site to be analysed by one laboratory to minimise potential inter-laboratory analytical bias. Nevertheless, there are some ambiguities on whether the samples have been processed freshly on arrival, or stored in a laboratory fridge or freezer for processing at a later date. The significance of understanding the stability of analytes in wastewater has been highlighted by previous stability studies. Holton et al. have revealed a degradation rate of antimicrobials (AAs) ranging from 10 to 60% after 24 h under refrigerated conditions [11]. Wen et al. have previously examined the stability of 17 common antiviral drugs in both sewage and sewer reactors, among which 9 were recommended as appropriate biomarkers for WBE with less than 20% degradation [12]. Study on the stability of 24 pharmaceutical biomarkers in a pilot sewer system simulating the conditions of real sewers has reported 17 biomarkers as stable [13].

Due to the diverse and dynamic nature of wastewater, an efficient and nonbiased extraction of total genomic bacterial DNA from the complex wastewater samples is a key first step for downstream molecular biology analysis such as quantitative PCR and metagenomic sequencing. Previous study has reported differences in bacterial community composition in human fecal samples between freshly extracted DNA and after freeze storage [14]. So far, studies focused on the effect of freezing and frozen storage on the chemical and biological properties of samples are mainly reported in the food domain [15]. The stability of AAs and ARGs in wastewater during freezing and frozen conditions is yet to be studied. With this research gap, this paper intends to explore some complexities of the analytics when linking biology and chemistry in the environmental AMR field.

This study is a continuation of a previous study about the stability of AAs at room temperature (18–21 °C) and refrigerator condition (8–14 °C) to evaluate the degradation of compounds during the 24-h composite sampling process [11]. In this study, the authors reported variations in stability between AAs as well as the two temperature conditions and concluded that degradation, adsorption, and biotransformation could be responsible for the variation in stability. To better align with the typical framework of environmental AMR surveillance, which involves the continuous collection of wastewater samples over several months or years, the present study was conducted to evaluate the stability of antimicrobials and ARGs in influent wastewater during a 52-week period of freezing storage at − 20 °C. Overall, this study is aimed to serve as a reference for standardisation and implementation of regional/national environmental AMR surveillance focussing on both chemical and biological targets.

## Methods and materials

### Chemicals

A total of 20 AAs and 11 major metabolites were targeted in the present study. The selected AAs covered a wide range of antibiotic classes including β-lactams, sulfonamides and trimethoprim, macrolides and lincomycin, quinolones, azoles, cyclines, tuberculosis (TB) drugs, and antiretrovirals. A total of 12 stable isotope–labelled internal standards (ISTD) were used for the quantification of the target compounds. A full list is provided in Table 1. More details on the chemical information can be found elsewhere [16]. Analytical standards and deuterated standards were purchased from Sigma-Aldrich (Gillingham, UK), TRC (Toronto, Canada), LGC (Middlesex, UK), or MCE (Cambridge, UK). HPLC-grade methanol (MeOH) and water were purchased from VWR (UK) and formic acid (> 95%) was purchased from Sigma-Aldrich, respectively. Stock solutions were prepared in MeOH at 1.0 mg/mL and mixed working solutions were diluted from the stock solutions to a final concentration of 1.0 mg/L in methanol. Stock and working solutions were stored in a − 20 °C freezer.

**Table 1:** Target antimicrobials, ARGs (antibiotic resistance genes), and abbreviations, ordered by drug classes

Drug class	Compounds/genes	Parent/metabolite	Abbrev	Drug class	Compounds/genes	Parent/metabolite	Abbrev
Sulfonamide and trimethoprim	Sulfadiazine	Parent	SDZ	Quinolone	Ofloxacin	Parent	OFX
	Sulfapyridine	Parent and active metabolite	SPY		Ciprofloxacin	Parent and active metabolite	CIP
	Sulfamethoxazole	Parent	SMX		Desmethyl-ofloxacin	Metabolite	dmOFX
	Sulfasalazine	Parent (prodrug)	SLZ		Ofloxacin-d3*	-	OFX-d3
	Trimethoprim	Parent	TMP		Ofloxacin desmethyl-d8*	-	dmOFX-d8
	N-acetyl sulfadiazine	Metabolite	aSDZ		*qnrS*	-	-
	N-acetyl sulfapyridine	Metabolite	aSPY	β-Lactam	Amoxicillin	Parent	AMOX
	N-acetyl sulfamethoxazole	Metabolite	aSMX		Amoxicilloic acid	Metabolite	AMXa
	Sulfamethoxazole-d4*	-	SMX-d4		Flucloxacillin	Parent	FLX
	Sulfasalazine-d4*	-	SLZ-d4		Cefalexin	Parent	LEX
	Trimethoprim-d9*	-	TMP-d9		Ceftriaxone	Parent	CRO
	*sul1*	-	-		Amoxicillin-d4*	-	AMOX-d4
Macrolide and lincosamide	Clarithromycin	Parent	CLR		Cefalexin-d5*	-	LEX-d5
	Clindamycin	Parent	CLI		*blaCTX-M*	-	-
	Erythromycin	Parent	ERY	Cycline	Oxytetracycline	Parent	OTC
	N-Desmethyl erythromycin	Metabolite	dmERY		Tetracycline	Parent	TET
	N-Desmethyl clarithromycin	Metabolite	dmCLR		Tetracycline-d6*	-	TET-d6
	N-Desmethyl clindamycin	Metabolite	dmCLI		*tetW*	-	-
	Clarithromycin-d3*	-	CLR-d3	Antiretrovirals	Emtricitabine	Parent	FTC
	Erythromycin-13C,D3*	-	ERY-13C		Lamivudine	Parent	3TC
	*ermB*	-	-	TB drug	Isonicotinic acid	Metabolite	INa
Azole	Metronidazole	Parent	MTZ		5-Hydroxy-pyrazinoic acid	Metabolite	hPZA
	Ketoconazole	Parent	KTC		Isoniazid-d4*	-	INH-d4
	Hydroxy-metronidazole	Metabolite	hMTZ	Other genes	*intI 1*	-	-
	Metronidazole-d4*	-	MTZ-d4		*16S rRNA*	-	-
	Ketoconazole-d3*	-	KTC-d3				

^*^Internal standards

### Stability study

Three litres of wastewater influent samples collected from a WWTP in the South West England was used as testing water. Flow-proportional composite sampling strategy was adopted by using ISCO 3700 auto-sampler to collect 15 mL of the influents at regular 15-min time intervals for 24 h. Samples were transported to the laboratory on ice on the day of collection. Once received, the wastewater was homogenised and 50-mL aliquots were prepared in triplicate according to 9 time points (*t* = 0 day, 3 days, 1, 4, 8, 13, 26, 39, and 52 weeks) for chemical and gene analyses, respectively. For chemical analysis only, each aliquot was spiked with internal standard at a final concentration of 1 μg/L, shaken, and left to partition for 30 min. All sample bottles were then stored at − 20 °C immediately (except for day 0) and defrosted according to different time points.

### Sample analysis

For AAs’ analysis, fresh sample (day 0) or the defrosted samples were processed via solid-phase extraction (SPE) following sample preparation protocol developed by Holton et al. [16]. Briefly, 50 mL of samples was filtered through a GF/F glass fibre filter (0.7 μm, Whatman) and the filtrates were loaded under vacuum onto pre-conditioned Oasis HLB cartridges (60 mg, Waters, UK) at 5.0 mL/min. The cartridges were conditioned and equilibrated with 2 mL of MeOH, followed by 2 mL of HPLC water under gravity. After loading, cartridges were dried under vacuum and stored in a − 20 °C freezer for elution at a later date. AAs are considered stable stored in a cartridge at − 20 °C [17]. Analytes were eluted using 4 mL MeOH under gravity and the eluate was collected in silanised vials (Thermo Scientific, UK) before evaporating to dryness. A reconstitution solution of 500 μL of 80:20 H_2_O:MeOH was used to re-suspend the resulting residues and final solution was transferred to injection vials (Waters, UK).

Liquid chromatography-mass spectrometry (LC–MS/MS) was performed using a Waters ACQUITY UPLC™ system coupled to a Xevo TQD-ESI Mass Spectrometer. Briefly, a reverse-phase BEH C18 column with Acquity column in-line 0.2-μm pre-filters (Waters, Manchester, UK) was used to separate analytes under a 19-min mobile phase gradient using 95:5 H_2_O:MeOH with 0.1% formic acid (mobile phase A) and 100% MeOH (mobile phase B). Mass spectrometry was performed via fully targeted multiple reaction monitoring (MRM), involving two MRM transitions per analyte and one per ISTD. Detailed information on chromatographic separation as well as mass spectrometry parameters can be found in a previous paper [16].

For target gene analysis, fresh sample or the defrosted samples were filtered using a vacuum filtration apparatus through a 0.2-μm filter unit (Nalgene, ThermoFisher, UK). Genomic DNA was extracted from the membrane using bead-beating method provided by FastDNA Spin kit for Soil (MP Biomedicals, UK) and DNA yield was quantified via Qubit 4.0 (Invitrogen, UK). Once extracted, DNA samples were kept at − 20 °C for downstream qPCR analysis. The concentration of all DNA samples was adjusted to 30 ng/μL prior to qPCR analysis. qPCR for each ARG was analysed via AriaMx Real-Time PCR system (Agilent Technologies, UK). Details on primers and qPCR protocol can be found elsewhere [18].

### Calculations/statistics

For chemical analysis, the stability of each analyte was determined by the relative change of concentration over the 52-week freezing period against day 0. For gene analysis, the overall variation of specific gene copy was presented as coefficient of variance (CV) to indicate how closely the data is clustered around the mean value. The average of triplicate samples was calculated for each time point and the error bars represented the standard deviation. Analyte concentrations were provided as ng/L or μg/L using TargetLynx software (Waters Lab Informatics, UK); gene concentrations (absolute abundance) were provided as copies/L by AriaMx qPCR software (Agilent Technologies, UK). Normalisation to bacterial *16S rRNA* for each ARG was expressed as the relative abundance. OriginPro 2019 was used to draw histogram and line graphs. Mean and standard deviation calculations were performed with Microsoft Excel 2016. One-way analysis of variance (ANOVA) and Pearson’s correlation analysis were performed using OriginPro 2019.

## Results

### Stabilities of antimicrobials

**Fig. 1:**
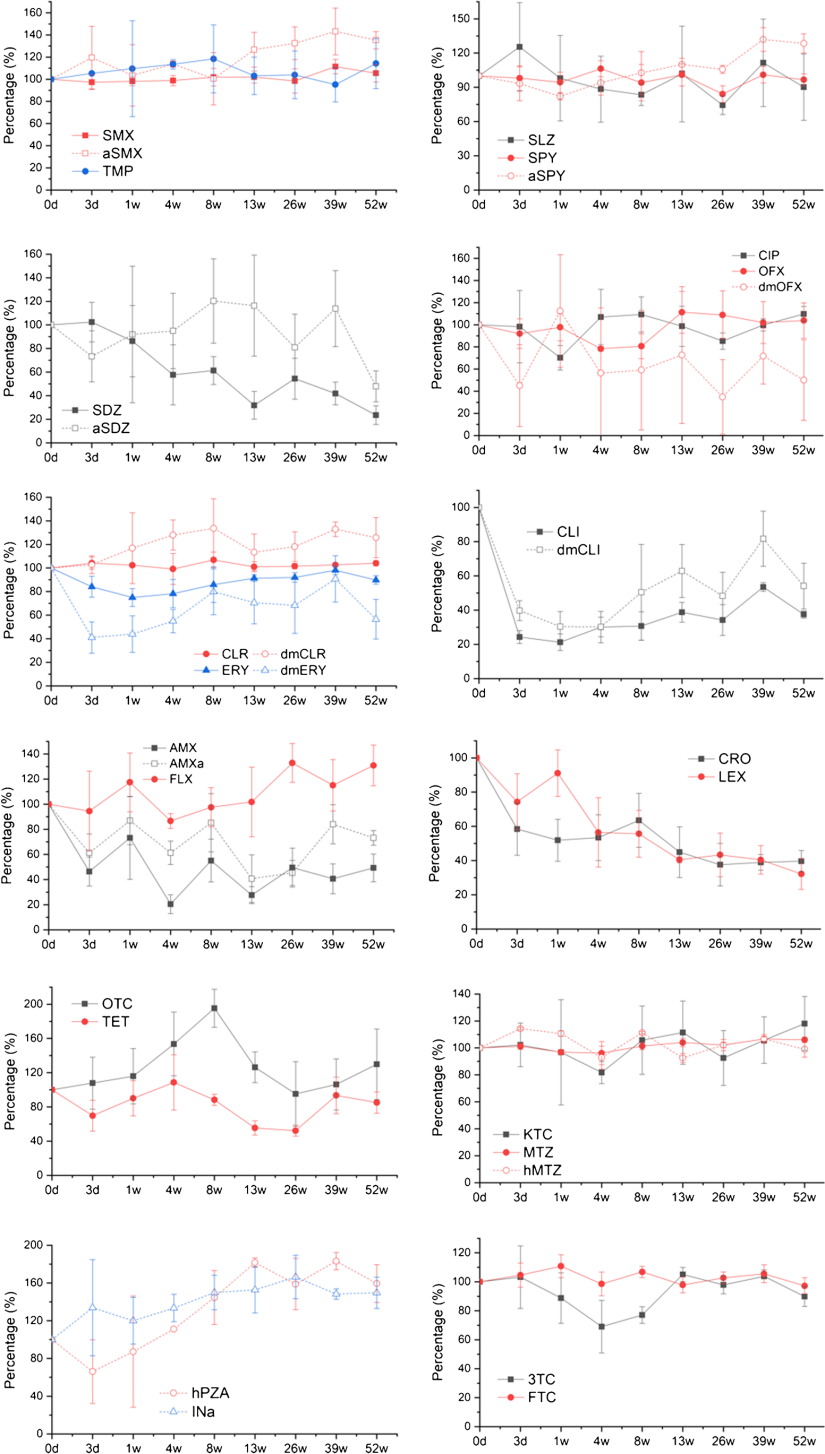
Antimicrobials stability across 1-year freezing period. Results are presented as percentage variation against the initial (day 0) concentration. Standard deviation error bar, *n* = 3

Figure 1 shows the stability results of all antimicrobials across the 52-week freezing period. Twenty-one out of the 31 compounds were generally stable throughout the freezing period, with concentration (mean value) varied less than 30% in comparison to the initial concentration in fresh sample. Among which sulfonamides and trimethoprim, macrolides, quinolones, azoles, and antiretrovirals were largely stable, while β-lactams displayed an approximate 55–33% decrease in concentration. In particular, clindamycin and its desmethyl metabolite exhibited an average of 66% and 50% reduction, respectively, in frozen condition. For those compounds which displayed variations throughout the freezing period, the largest decrease was observed between the fresh and first frozen samples (day 3), suggesting that the loss in concentration was independent of freezing period. No statistically significant differences were observed between fresh and frozen samples, or among samples at various freezing stages. Raw concentration data of individual antimicrobial at different time points are provided in supporting information (SI) Figure S1.

#### Sulfonamides and trimethoprim

Except for SDZ, parent compounds of sulfonamides (SLZ, SMX, and SPY) and trimethoprim were very stable under freezing condition, showing an average of 9 ± 6 to 13 ± 9% variation in comparison to the initial concentration (Fig. 1). Metabolites showed greater variability at 14 ± 11% for aSPY, 20 ± 15% for aSDZ, and 22 ± 15% for aSMX. Interestingly, SDZ showed a gradual decrease throughout the freezing period, dropped by 14% after a week, then a further 36% (weeks 4–39) and 27% (week 52). Only 23% of the initial SDZ was recovered at the end of the study.

**Fig. 2:**
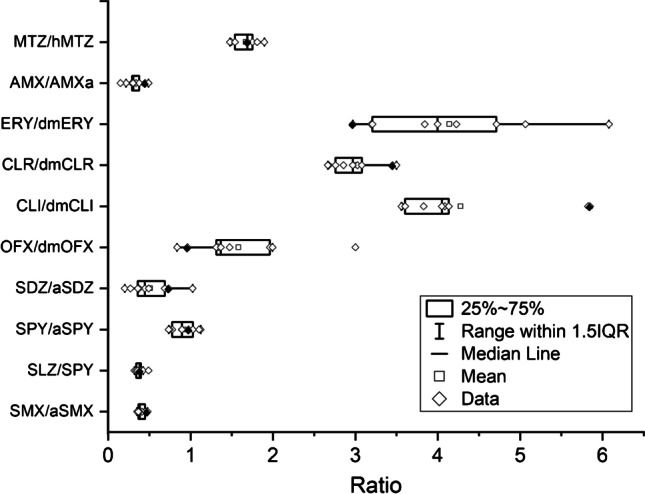
Box chart showing parent/metabolite ratio throughout the stability study. Black dots indicate the ratio in fresh sample (day 0)

Results for the parent/metabolite ratio also reflected the impact of freezing storage. SMX/aSMX, SLZ/SPY, and SPY/aSPY ratios were consistent throughout the study, recorded at 0.40 ± 0.05, 0.38 ± 0.05, and 0.92 ± 0.15, respectively (Fig. 2). SDZ/aSDZ ratio varied as a result the decreasing concentration of SDZ at 0.51 ± 0.26.

#### Quinolones

Ciprofloxacin and ofloxacin were almost fully recovered (97 ± 14%) from the frozen samples (Fig. 1), independent of the freezing time. In comparison, the desmethyl metabolite of ofloxacin displayed variations between different time points, ranging from 35 to 112% with no clear recovery patterns. Initial OFX/dmOFX ratio in fresh sample was 0.96 and the value varied between 0.84 and 3.00 (CV = 42%) in all frozen samples (Fig. 2).

#### Macrolides and lincomycin

Consistent and stable recoveries were observed for both CLR and ERY, recorded at 103 ± 2% and 87 ± 8%, respectively (Fig. 1). In comparison, their desmethyl metabolites showed greater variabilities at 121 ± 11% and 63 ± 17%, respectively. dmERY showed a random recovery pattern, decreased by approximate 53% when freezing for a month, then increased by 31% at longer freezing period and decreased again by 21% for the last time point. Distinct concentration losses were observed for CLI and dmCLI. The largest loss was between the fresh and first frozen samples (day 3), dropped by 76% and 60%, respectively. Average recoveries for the remaining frozen samples were 34 ± 10% and 50 ± 17% for CLI and dmCLI, respectively. Although CLI and dmCLI decreased rapidly in concentration once frozen, ratios of CLI/dmCLI were generally consistent across all frozen samples, averaged at 4.28 ± 0.91 (Fig. 2). CLR/dmCLR and ERY/dmERY recorded at 3.00 ± 0.31 and 4.14 ± 1.01, respectively, showed no clear impact of freezing storage.

#### β-Lactams

Only 45–67% (average value) of the starting concentration was recovered in β-lactams (except for FLX) (Fig. 1). For the two cephalosporins, CRO decreased rapidly once froze and stayed consistent in all frozen samples (49 ± 10% recovery). By contrast, the concentration of LEX decreased gradually with increasing freezing period and 32% was recovered in the last frozen sample. AMX and its metabolite AMXa showed similar and fluctuating trends, with parent compound decreased by 55 ± 16% and AMXa decreased by 33 ± 18%. Ratio of AMX/AMXa was recorded at 0.32 ± 0.11 (Fig. 2). FLX is the only β-lactam which stayed stable (110 ± 17% recovery) across all time points.

#### Tetracyclines, azoles, TB drugs, and antiretrovirals

OTC and TC were generally recovered from the frozen samples, recorded at 129 ± 32% and 80 ± 20% recoveries, respectively (Fig. 1). Azoles, MTZ, hMTZ, and KTC were also very stable, showing a recovery of 102 ± 4%, 104 ± 8%, and 102 ± 11%, respectively. Similarly, MTZ/hMTZ stayed consistent at 1.67 ± 0.14 throughout the study (Fig. 2).

Parent compounds of TB drugs were below LOD and two metabolites, hPZA and INa, showed increasing trends in concentration with freezing period (Fig. 1). In fact, average recoveries of the two compounds in frozen samples were the highest compared to other compounds, recorded at 136 ± 44% and 144 ± 14%, respectively. The two antiretrovirals, 3TC and FTC, were mostly recovered (92–107%) (Fig. 1).

### Variations of ARGs

**Fig. 3:**
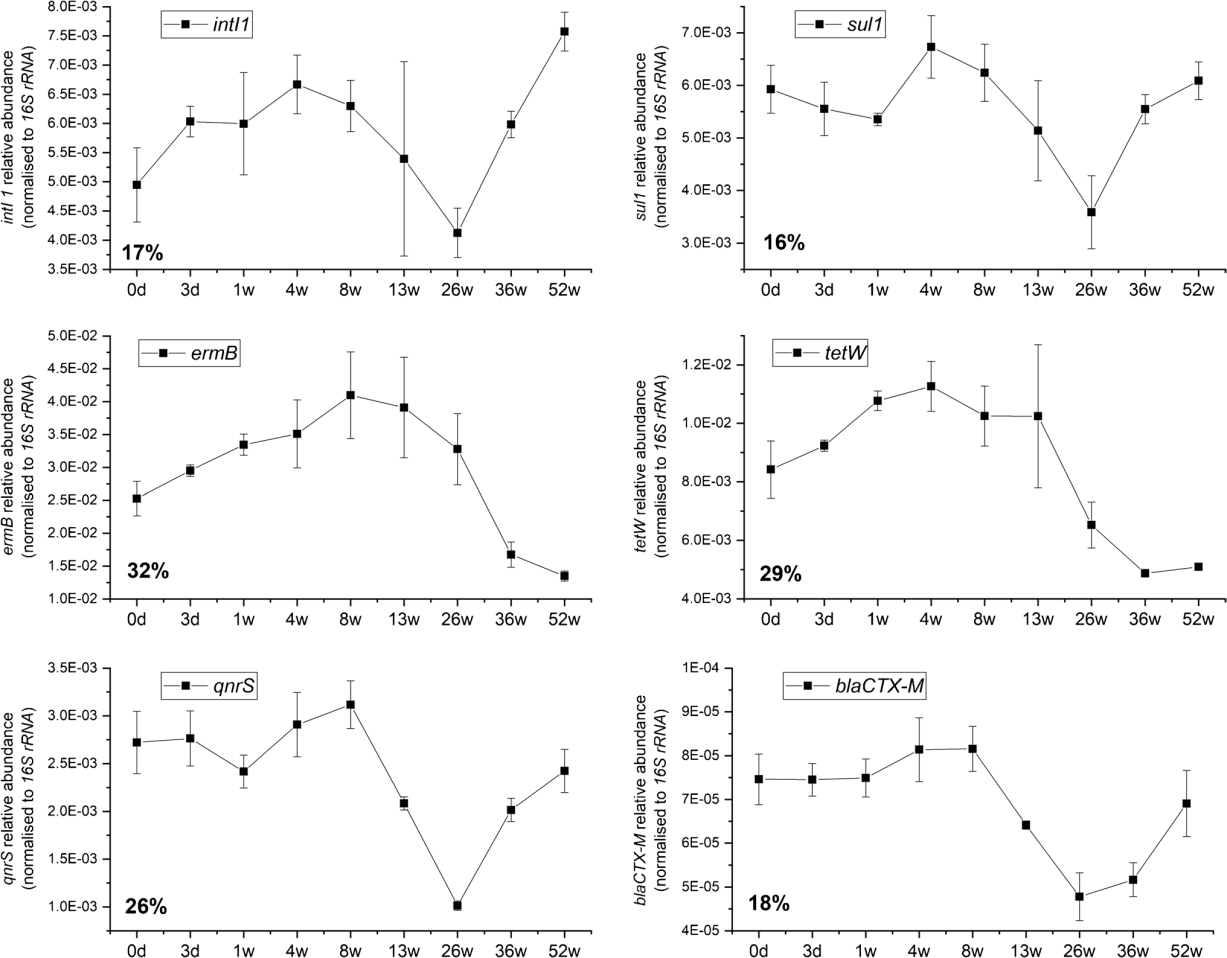
Variation of the relative abundance of target genes (normalised to *16S rRNA*) across 1-year freezing period. Percentage indicates the coefficient variance for each gene

Concentration of each gene is expressed as gene copy per litre and CV is calculated to indicate the overall variations across fresh and all frozen samples. As the most abundant target gene, *16S rRNA* showed the least variation between fresh and frozen samples, ranging from 2.08 ± 0.41 × 10^11^ copies/L to 3.17 ± 0.44 × 10^11^ copies/L with 15% variations observed (Figure S2). To minimise the variation in ARG caused by the background bacterial community, Fig. 3 shows the relative abundance of target genes (normalised to *16S rRNA*) across 1-year freezing period. Lowest and highest variations were observed for *sul1* and *ermB* genes at 16% and 32%, respectively. Most of the genes showed fluctuating trends with no clear patterns, where *ermB* and *tetW* dropped by approximately 50% at the last two time points (Fig. 3). No significant differences were observed between the fresh and corresponding freeze-stored samples. It should be mentioned that the gene relative abundance data showed little variation (3–11%) when log 10-transformed.

In general, the relative abundance of *intI1*, *sul1*, and *blaCTX-M* showed lower variabilities (16–18%) in comparison to *ermB*, *tetW*, and *qnrS* (26–32%). For *ermB*, changes did not exceed 20% of the initial concentration over 26-week freeze period, but further storage resulted in decrease below 55% of initial value. Gene concentration dropped by two times from 8.23 ± 0.83 × 10^9^ copies/L to 4.87 ± 0.83 × 10^9^ copies/L at week 36 and maintained at lower level (3.51 ± 0.32 × 10^9^ copies/L) at the end of the study (Figure S2). Similar pattern was observed for *tetW*, where concentration reduced from 1.93 ± 0.09 × 10^9^ copies/L at 26 weeks to 1.26 ± 0.06 × 10^9^ copies/L at 36 weeks. No consistent difference in the relative abundance of *intI 1* or *sul1* was observed between the fresh and corresponding freeze-stored samples. These two genes displayed comparable gene abundance and variability, recorded at 1.47 ± 0.26 × 10^9^ copies/L and 17% variation for *intI1*, and at 1.40 ± 0.27 × 10^9^ copies/L and 16% variation for *sul1*. Further analysis of qPCR data showed that the variations of *intI1* and *sul1*, *tetW* and *ermB*, and *qnrS* and *blaCTX-M* display significant correlations (Pearson’s* r* = 0.76, *p* < 0.05; *r* = 0.84, *p* < 0.01; *r* = 0.93, *p* < 0.01; respectively).

## Discussion

### Effect of freezing and frozen storage on antimicrobials

Most of the AAs (66%) detected were stable after 1-year freeze storage. In general, the effect of freezing wastewater samples prior to SPE analysis can be divided as follows: no effect (80–120% recovery), representative AAs are sulfonamides, macrolides, quinolones, tetracyclines, azoles, and antiretrovirals; moderate effect (60—80% recovery), where 55% of metabolites are subject to moderate effect; and major effect (less than 60% or above 120% recovery), representative AAs are β-lactams, TB drugs, clindamycin and its desmethyl metabolite.

For those compounds that were less affected by freeze storage, the low variability in concentration could be partially attributed to a good quantification method, where the loss of compounds can be corrected for by the equal loss of the deuterated isotopic ISTD. This factor can be excluded in this study as consistent peak areas were observed for all ISTDs over the entire freeze period with no statistical difference, suggesting that the variation of AAs is mainly linked to their stability. A box plot showing the peak areas of ISTDs can be found in SI Figure S3. Representative good examples are SMX and aSMX; SLZ; TMP; TET, CLR, and dmCLR; ERY; MTZ and hMTZ; and KTC and OFX. On the contrary, the decrease in concentration for certain AAs was mainly due to the loss of the AA itself. For instance, metabolites of both ERY and OFX displayed a 37% reduction (on average) in comparison to their initial concentration; and LEX reduced gradually over freeze time by 46% (on average). Several compounds, i.e. SPY, aSPY, aSDZ, CIP, OTC, EMB, FLX, 3TC, and FTC, were largely recovered from the frozen samples, albeit not using the corresponding deuterated isotopic ISTD. This indicates that these compounds are highly stable under frozen condition and suitable for long-term storage. There is lack of information on the effect of freezing with time on the stability of antimicrobials in wastewater specifically; previous study has mainly focused on foods of animal origin such as chicken, beef and pork. Cooling and freezing for 4 weeks could slightly reduce the antibiotic residues in beef [19], while CIP completely disappeared and OTC was reduced by 32% in chicken meat samples after freezing for 12 months [20]. The inconsistencies observed regarding stability of AAs in frozen samples may suggest that degradation pathways differ across various matrices.

Wastewater represents a complex, non-sterile system where enzymatic metabolism may occur even at freezer temperature of − 20 °C. A negative correlation was observed for SDZ and aSDZ (*r* =  − 0.70, *p* = 0.08), albeit not significant. This was further evidenced by a decreasing SDZ/aSDZ ratio starting from 1 week of freezing. Previous studies have reported a variety of SDZ-degrading bacteria (e.g. *Arthrobacter*, *Microbacterium*, *Methylobacterium*, and *Paracoccus*) isolated from WWTP activated sludge [21, 22]; the decreasing in SDZ and increasing in aSDZ concentration observed may indicate a potential formation of the transformation product under freezing condition. This could possibly be the case for the two TB drug metabolites, hPZA and INa, as both experienced a gradual increase in concentration during the freezing period.

β-Lactam represented a drug family which is highly affected by freeze storage, except for a penicillin FLX, the remaining compounds AMX, AMXa, two cephalosporins CRO and LEX were poorly recovered from the frozen samples with an average of 54% recovery recorded. This drug family is well-known for their instability in natural aquatic environments, where chemical hydrolysis or cleavage of the unstable β-lactam ring by β-lactamases could be expected [23]. Although no previous data are available with regard to β-lactam stability in wastewater at − 20 °C, the degree of degradation was reported up to 30% for β-lactams in plasma samples when stored at − 20 °C for 4 days [24] and no degradation (less than 6%) when stored at − 70 °C for 8 months [25]. O’Brien et al. also reported a degradation rate up to 38.4% for ampicillin (closely related to amoxicillin) in tissue samples when stored at − 20 °C [26]. By contrast, AMOX retained stability within 4 weeks in milk samples when kept at − 20 °C [27]. It is interesting to note that concentration of the two cephalosporins, CRO and LEX, decreases continued progressively throughout the study, suggesting that longer storage time could lead to higher degradation rate. It is, therefore, recommended that for the quantification of β-lactams in wastewater, either lower storage temperature conditions (e.g. − 70 °C) or short storage period (within a week) at − 20 °C should be considered. CLI and its desmethyl metabolite exhibited distinctive pattern in the stability study and only 34–50% of the starting sample concentration was recovered. The largest decrease in concentration was observed between the first two time points (fresh vs frozen), suggesting that the initial loss was due to potential partitioning of analytes into solid phase during the cooling stage, when precipitates formed at the bottom prior to ice crystal growth [28]. This part was subsequently removed during SPE process. This is possibly also the case for the loss of dmOFX concentration, as its parent compound OFX was stable during the freezing period. Similar observation of 20–50% recovery was reported for CLI and dmCLI at both ambient and refrigerated temperatures [11]. This highlights the importance of recognising CLI and dmCLI stability during sample storage, where a consistent pre-treatment procedure should be followed.

While this study assesses the freezing effects on the stability of AAs and reports their corresponding recovery based on a 1-year observation, no statement could be made on a systematic correction factor for those significantly affected AAs, such as β-lactams, CLI, and dmCLI. This is mainly due to the dynamic and complex nature of wastewater and storage condition may vary in terms of sample transportation and preservation in different laboratories. Nevertheless, it is important to reflect the impact of long-term freeze storage condition after sampling on AAs.

### Effect of freezing and frozen storage on ARGs

The assessment of freeze storage of wastewater on ARG variation in the present study is based on the assumption that the cooling rate and ice formation inside biological cells were equal during freezing. The freezing process consists of cooling stage, phase change stage, and solidification stage [28]. For wastewater specifically, settleable particles could sink down to the bottom by weight quickly during the cooling stage, in which sample retains its liquid format prior to the ice formation. It is worth to mention that after thawing, samples were filtered through the membrane easier compared to the fresh sample, likely due to the change in microstructure of wastewater during the freezing process. Previous study on landfill sludge has suggested that the growing ice crystals could compress and destroy the floc structure of the sludge, which rendered the small particles agglomerate together to form large clusters [29]. Limited studies have addressed the effect of storage conditions on the stability of ARGs in wastewater samples. *Sul1*, *blaCTX-M*, and *blaTEM* have proven to be persistent without significant change in concentration at 4 °C for a month in wastewater influent [30]. By contrast, more studies have focused on the effect of freeze storage (− 20 °C or − 80 °C) on bacterial community structure in soil, animal, and human-associated samples [31–33]. It remains unclear whether the freeze–thaw process facilitates the selection of a certain groups of microorganisms (e.g. ARGs hosts) in wastewater, as controversial observations have been reported in previous studies. Bahl et al. have confirmed that freezing fecal samples prior to DNA extraction affects bacterial community structure expressed as *Firmicutes* to *Bacteroidetes* ratio [14], while the phylogenetic structure and diversity of communities were not significantly influenced by the storage temperature at − 20 °C in human fecal samples. Further metagenomic study is needed to clarify this issue.

The effect of freeze/thaw on the stability of extracted DNA was not considered a potential degradation source, as repeating freeze and thaw cycles were avoided during the analysis [34]. Most of the time, the extracted DNA is frozen at − 20 °C or − 80 °C. In this study, the extracted DNA was kept at − 20 °C for less than 3 months before thawing for the downstream qPCR analysis. While it is not as stable as storing DNA at lower temperatures, such as − 80 °C, − 20 °C is generally sufficient for short- to medium-term storage of genomic DNA [35]. Other potential influential factors include DNA extraction efficiency and insufficient mixing and handling of thawed wastewater/DNA samples prior to analysis. An internal standard, as commonly used in analytical chemistry, can be spiked in wastewater samples for future ARG stability monitoring. For instance, *Escherichia coli* strain transformed with an internal reference gene *molA* has been used to assess the matrix effect of wastewater on DNA extraction and qPCR determination for ARGs [36]. Furthermore, Crossette et al. reported using genomic DNA as internal standard for metagenomic quantification of ARGs in environmental samples [37].

### Implication on wastewater AMR surveillance

To fully understand AAs’ stability not only during storage but also during sampling, a previous study has been included here to compare degradations under different conditions [11]. Table 2 lists percentage degradation of AAs at ambient, refrigerated, and freeze storage conditions. Stability of AAs in the sewage system (ambient temperature), during the 24-h sampling (refrigerator temperature) and freeze storage (− 20 °C), is accounted to provide a comprehensive perspective when applying WBE tool. It should be mentioned that temperature control measure is required for the auto-sampler used during the 24-h composite sampling, for example ice packs could be introduced to cool sub-samples during collection [5]. As shown in Table 2, the overall degradation of AAs can be classified as low (< 30%), moderate (30–60%), and high (> 60%). Among all 32 AAs presented in samples, 38% showed low degradation; 22% showed moderate and 40% showed high degradations. Sulfonamides, macrolides, and antiretrovirals are generally stable across all conditions and can be considered appropriate biomarkers to reflect the actual community usage. On the contrary, OFX, dmOFX, and tetracyclines, which are frequently targeted in WBE studies, are subject to very high degradations. Similarly, azoles are very stable at freeze storage condition but degrade up to 61% at ambient and refrigerated conditions. Although reports on β-lactams in WBE study are limited, consistent degradations under all conditions have been observed. Worryingly, CLI and dmCLI showed the highest overall degradations among all AAs detected. Analysis of both parent and metabolite has been recently included in WBE studies to provide valuable community-wide AAs’ usage data and to verify potential misuse of AAs such as direct disposal [11, 38–40]. Together with Holton et al.’s observations, the extents to which storage conditions affect parent and metabolite compounds varied, such as CLI/dmCLI, ERY/dmERY, OFX/dmOFX, and SDZ/aSDZ. This could potentially bias the parent/metabolite ratio calculation and lead to an inappropriate selection of biomarker. As a data-driven research requiring full quantification, the above evidence clearly suggests the need for full appreciation of sampling, storage, and analysis linked uncertainties, also including stability study as a complementary aspect of the WBE tool as well as to optimise the analytical assays of AAs for easier laboratory routine. For instance, a correction factor could be potentially applied to correct for the amount of individual AA; or a well-chosen ISTD should be spiked prior to long-term storage to maintain stability. Having said that, residence time of AAs in the sewage system varies significantly with respect to community size; stabilities at refrigerated and freeze storage conditions are more likely to be accounted for to minimise the potential under- or over-estimation of AAs’ usage.

**Table 2:**
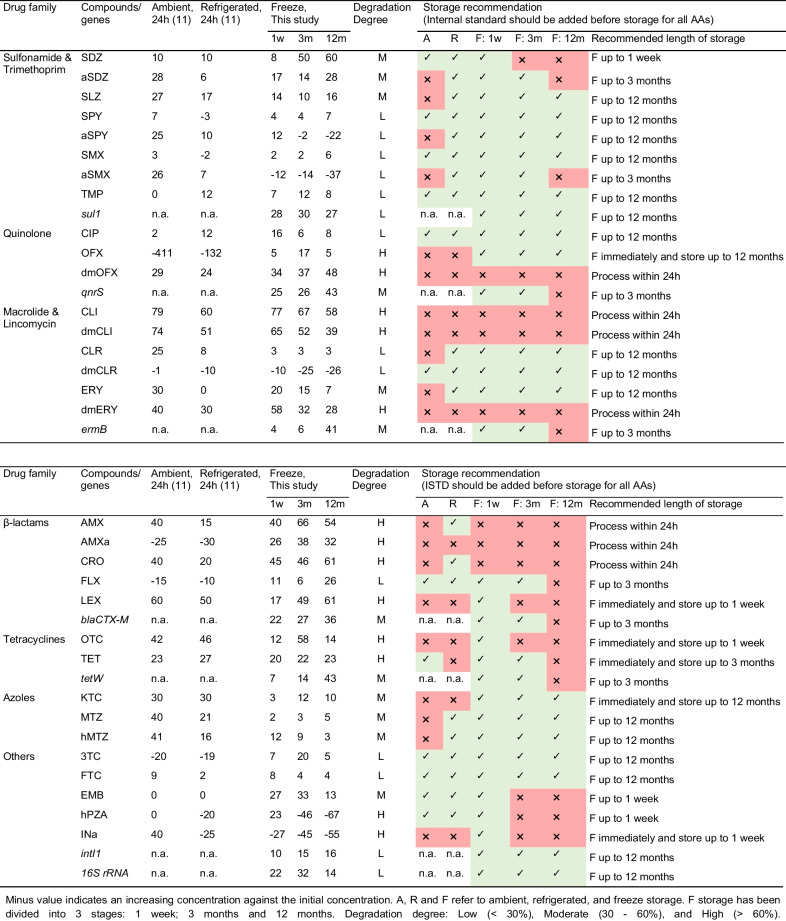
Percentage degradation of antimicrobials/ARGs at ambient (A, 20 °C, 24 h) (11), refrigerated (R, 11 °C, 24 h) (11), and freeze storage (F, − 20 °C) conditions

Minus value indicates an increasing concentration against the initial concentration. A, R, and F refer to ambient, refrigerated, and freeze storage. F storage has been divided into 3 stages: 1 week; 3 months, and 12 months. Degradation degree: low (< 30%), moderate (30–60%), and high (> 60%)

### Recommendations

According to Table 2, a testing laboratory should avoid unnecessarily extended storage times of wastewater samples, and thus, stability data of the ARGs at − 20 °C up to 3 months seems appropriate. Having said that, freezer space can be a limiting factor for the storage of large volume of wastewaters (particular in the case of intensive sampling campaign for weeks). The 3-month storage period could offer a sufficient turnaround time to facilitate logistics of laboratory routine. For chemical analysis, isotope-labelled internal standards should be added to samples on the same day after collection. The rate at which degradation occurs differs among various drug families and within the same drug family; researchers are suggested to refer to the analyte-specific storage recommendations as listed in Table 2. Results obtained from this study apply not only to wastewater influent but also potentially to less complex environmental samples (e.g. wastewater effluent and river water). Accurate environmental data would help to establish model-based evaluation tool and in turn to better predicting AA concentrations in various types of aqueous samples such as wastewater influent and effluent and river [41].

## Conclusion

This study examines the stability of AAs and variations of ARGs in a reference wastewater samples stored under frozen condition over a period of 12 months. The main conclusions are as follows:

- Sulfonamides, quinolones, macrolides, tetracyclines, azoles, and antiretrovirals were stable under frozen conditions, where β-lactams were poorly recovered (45—67%) from the initial concentration.
- Only 34–50% of the starting concentration was recovered for clindamycin and its desmethyl metabolite and the largest decrease was observed between the fresh and first frozen samples. This suggests that the initial loss may be due to the partitioning of analytes into solid phase during the cooling stage.
- Parent compounds were more stable compared to their metabolites under frozen conditions.
- Gene analysis showed very limited effect on *16S rRNA*, *sul1*, and *intI 1* and moderate effect on *ermB*, *qnrS*, and *blaCTX-M*.

In general, the storage of wastewater samples at − 20 °C is recommended for AAs and ARG analysis for easier laboratory routine. Researchers interested in looking for both or either antibiotic and ARGs in wastewater samples need to be aware of the stability issue for certain compounds in the sewage system, during sampling as well as laboratory storage. This is particularly important for data-driven studies such as WBE and AMR to inform public health and resistance development at community level.

### Supplementary Information

Below is the link to the electronic supplementary material.

Supplementary file1 (DOCX 1073 KB)
