# Supplementary material for: Assessment of the stability of antimicrobials and resistance genes during short- and long-term storage condition: accounting for uncertainties in bioanalytical workflows
Source: Anal Bioanal Chem. 2023 Aug 1;415(24):6027–38. doi: 10.1007/s00216-023-04874-6 (PMC10556160; doi:10.1007/s00216-023-04874-6)
Supplement: Supplementary file 1 — Supplementary file1 (DOCX 1073 KB) [file 216_2023_4874_MOESM1_ESM.docx]

**Supporting Information**

**Assessment of the stability of antimicrobials and resistance genes during short and long-term storage condition: accounting for uncertainties in bioanalytical workflows**

Like Xu^1^, Barbara Kasprzyk-Hordern^1,2,3^*

*^1^Department of Chemistry, University of Bath, Claverton Down, Bath BA2 7AY, United Kingdom*

*^2^Institute for Sustainability, University of Bath, Claverton Down, Bath BA2 7AY, United Kingdom*

*^3^Water and Innovation Research Centre, University of Bath, Claverton Down, Bath BA2 7AY, United Kingdom*

* Email address: [b.kasprzyk-hordern@bath.ac.uk](mailto:b.kasprzyk-hordern@bath.ac.uk)

| 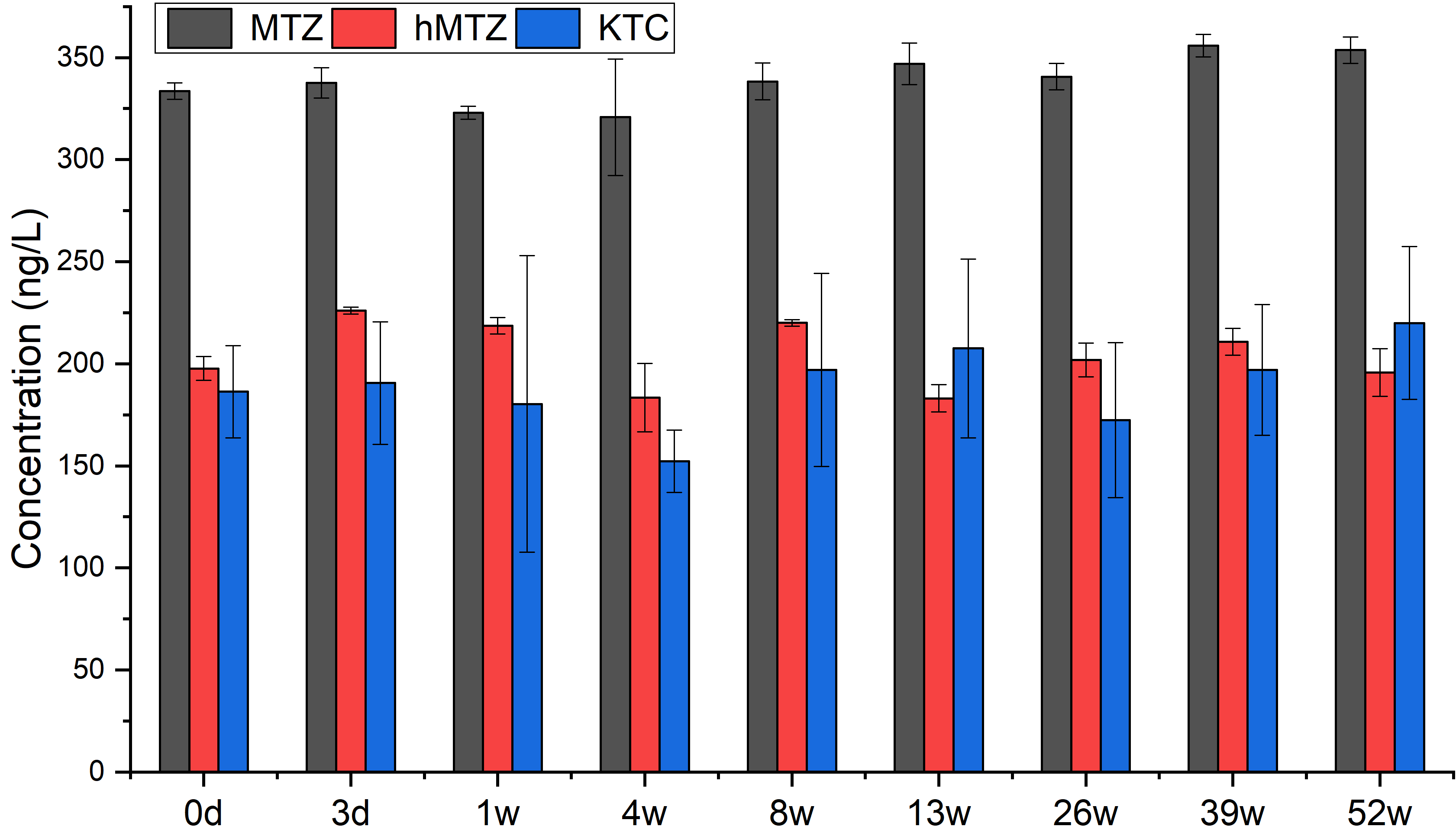 |
| --- |
| 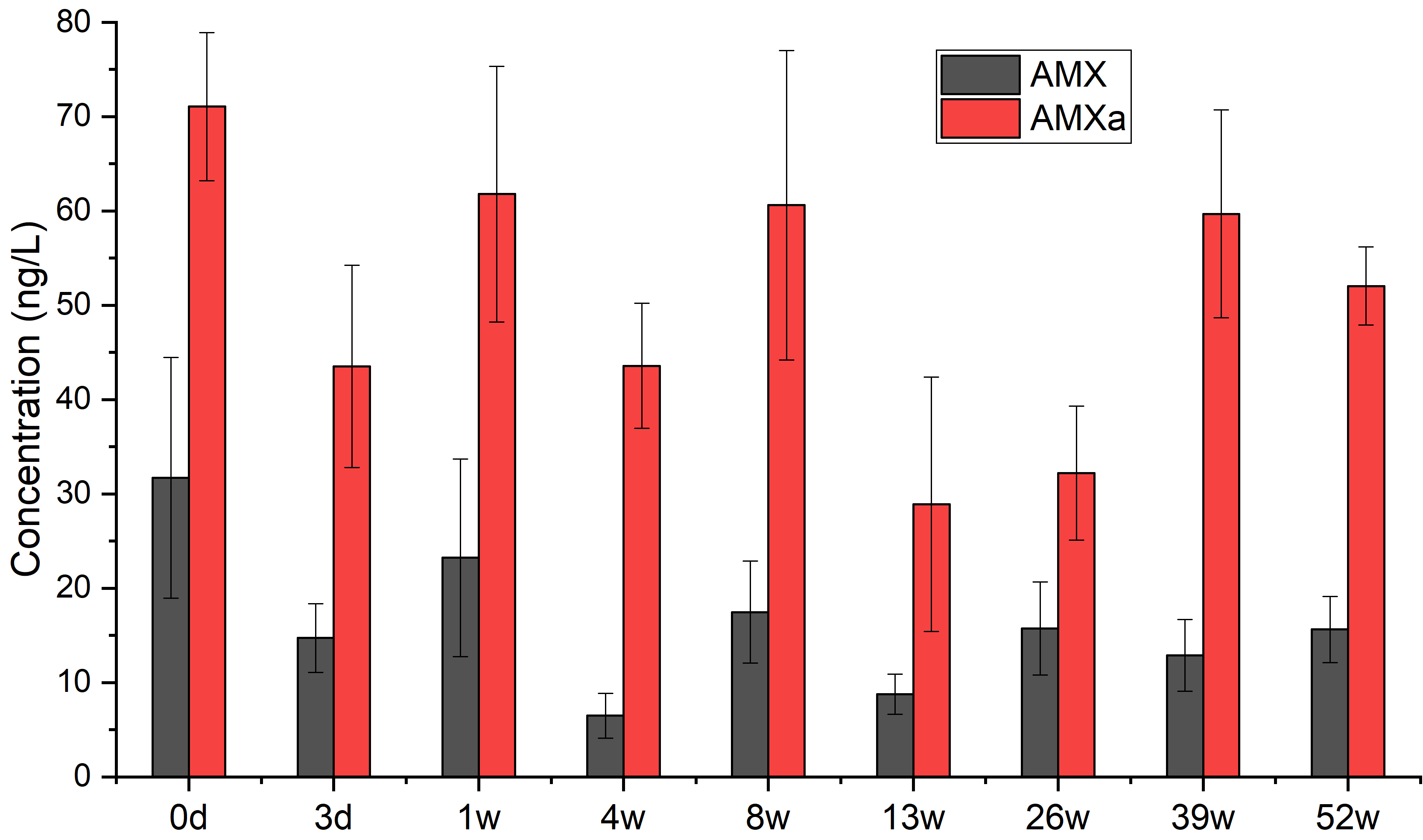 |
| 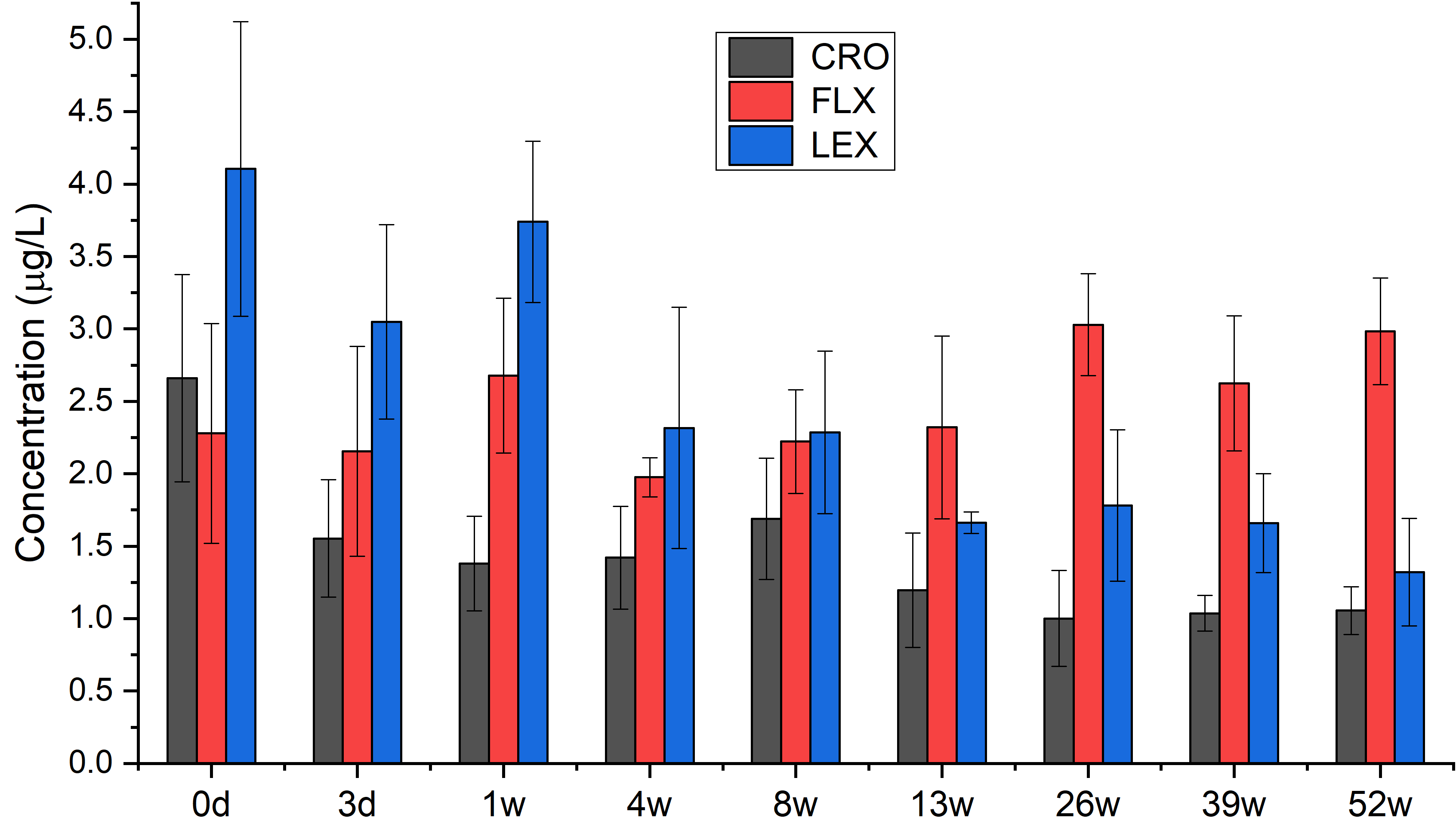 |
| 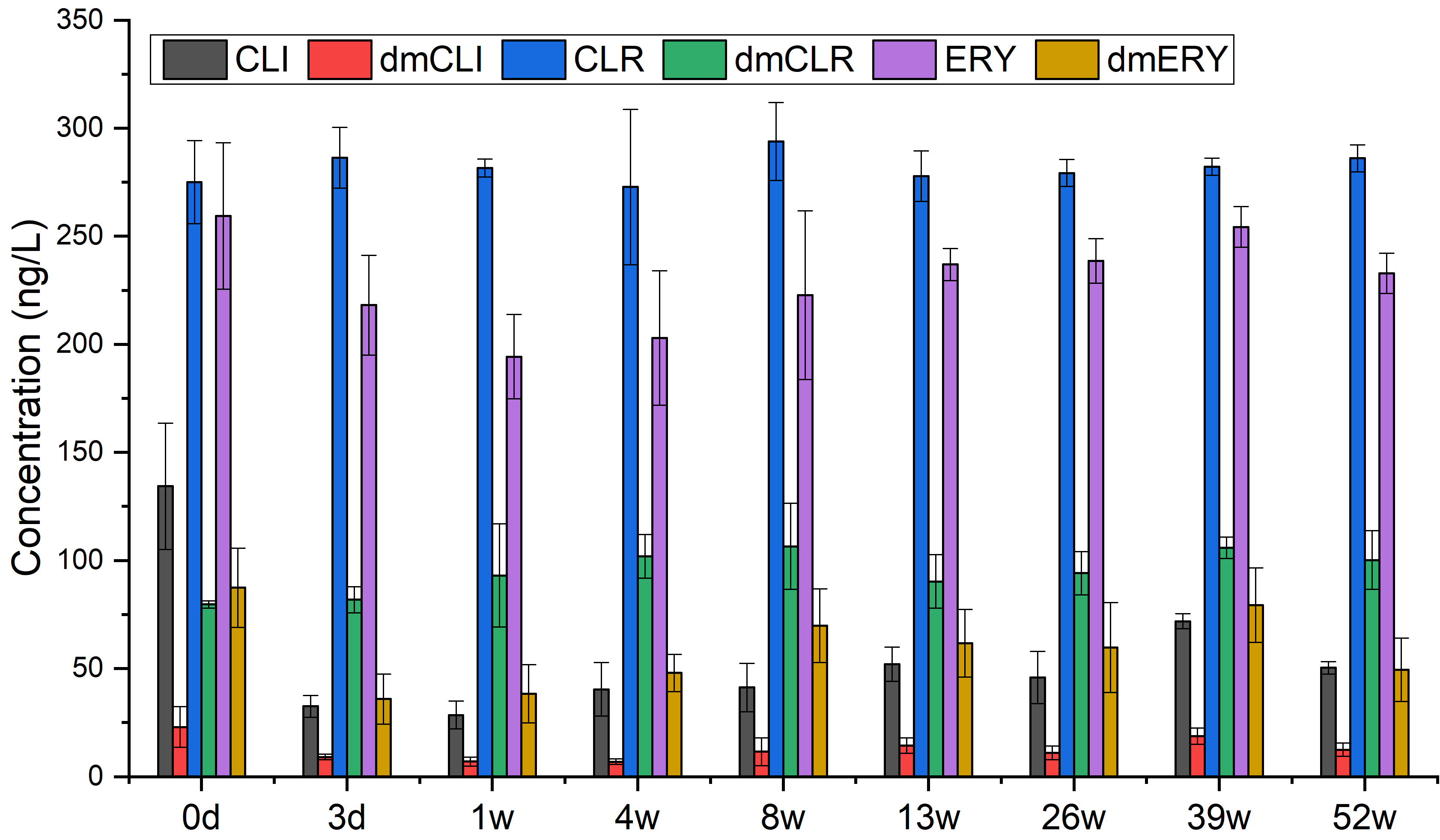 |
| 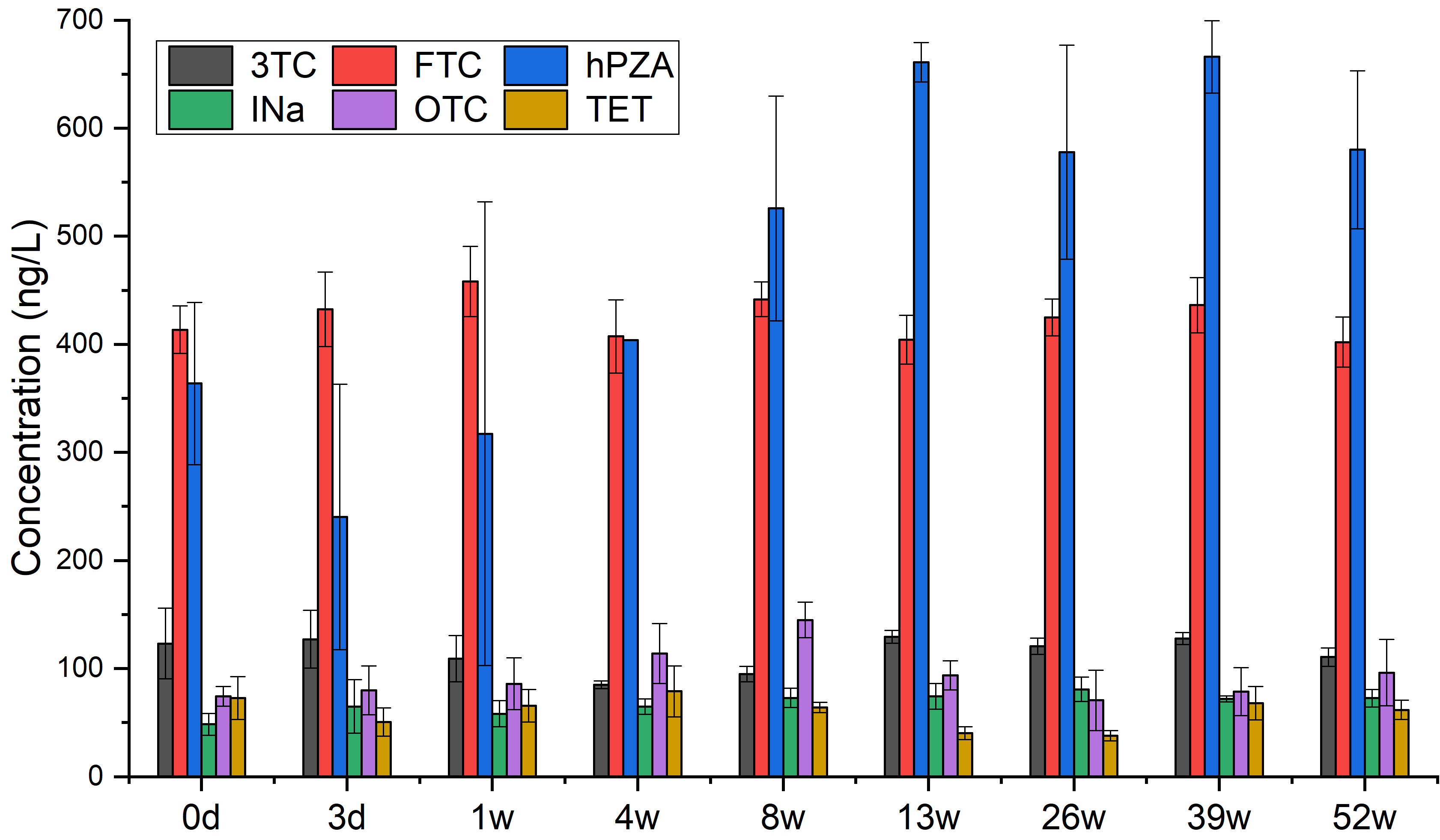 |
| 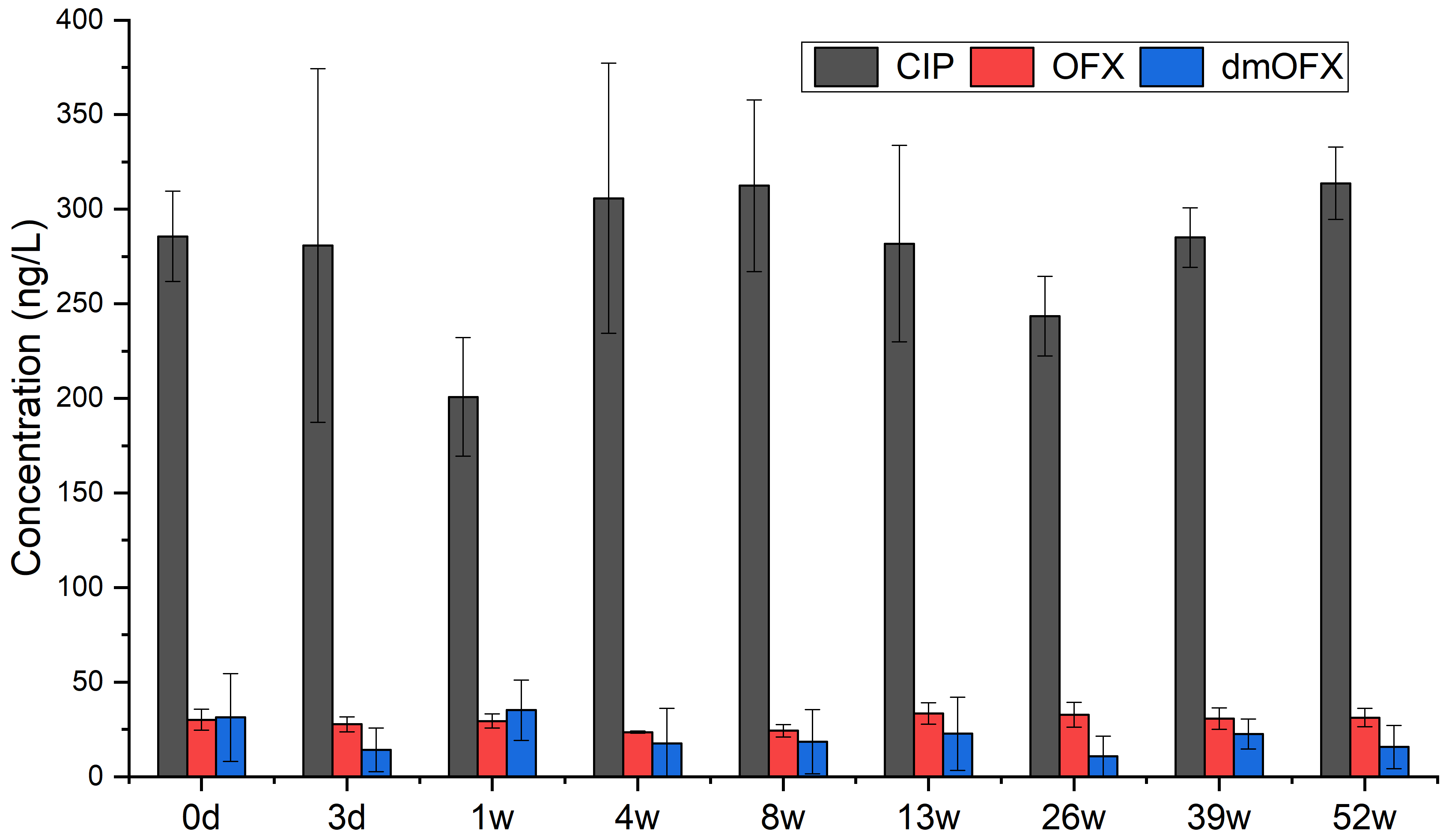 |
| 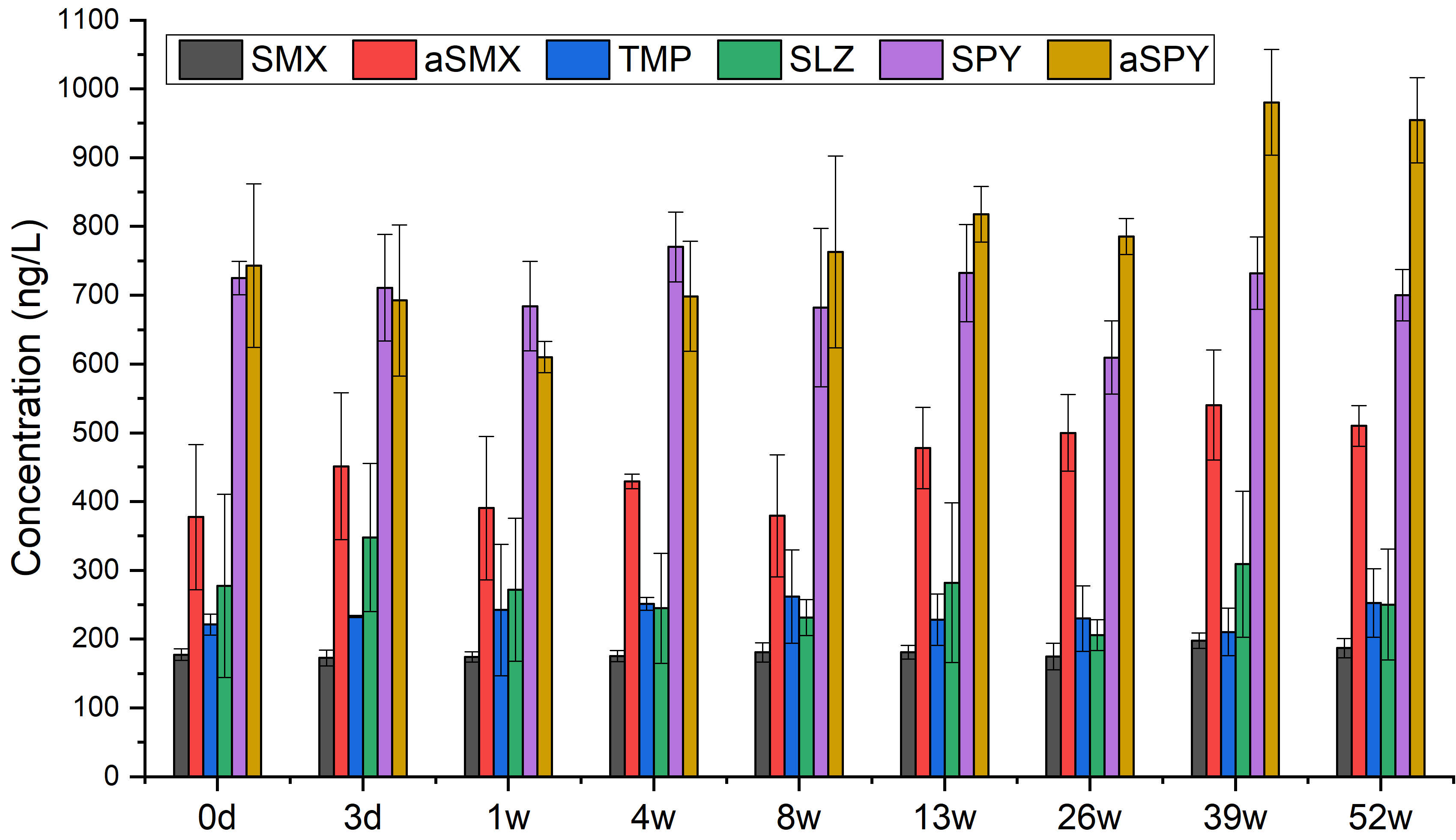 |
| 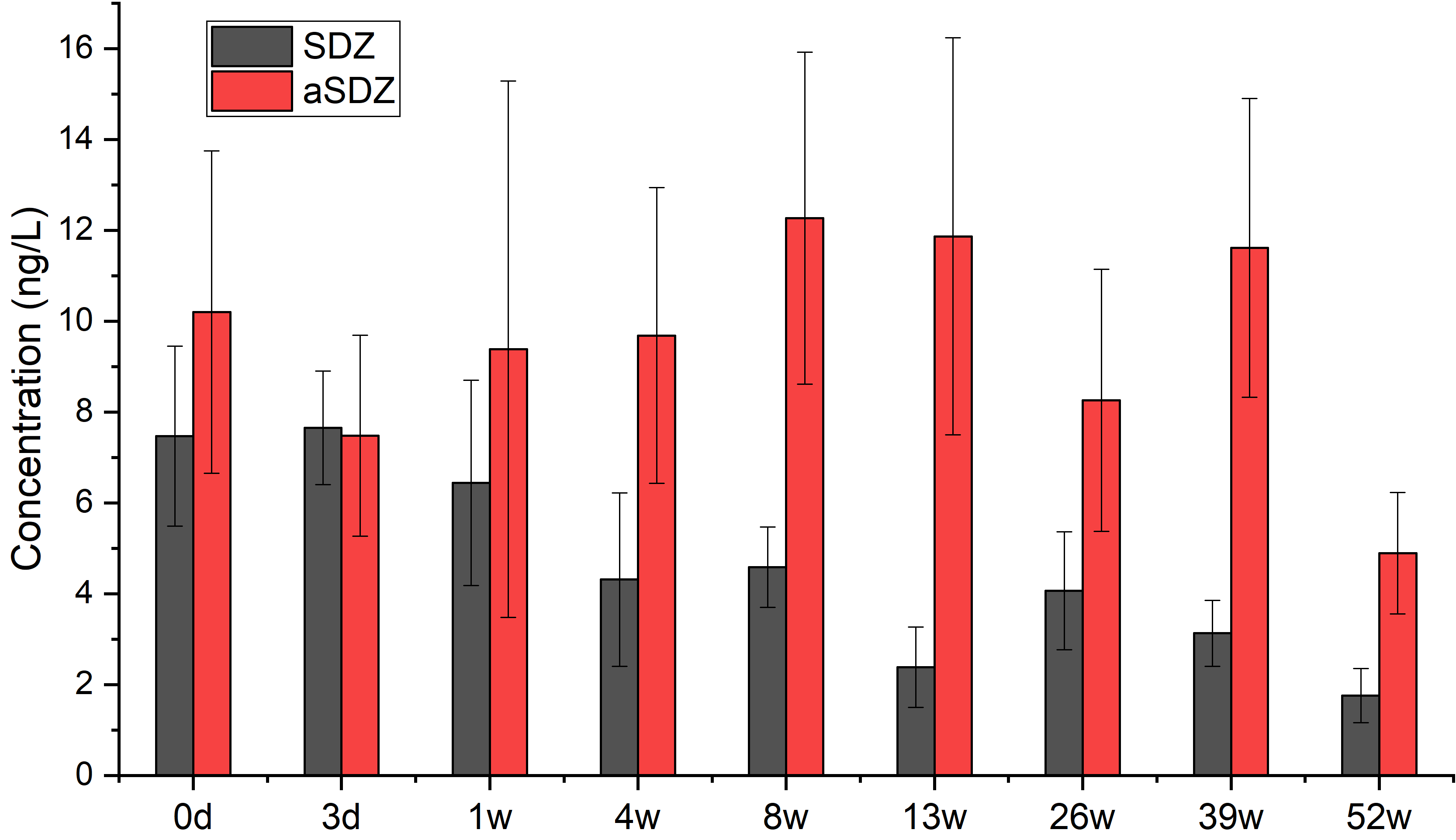 |
| Figure S1. Concentrations of antimicrobials detected in stability samples (n = 3). |

| 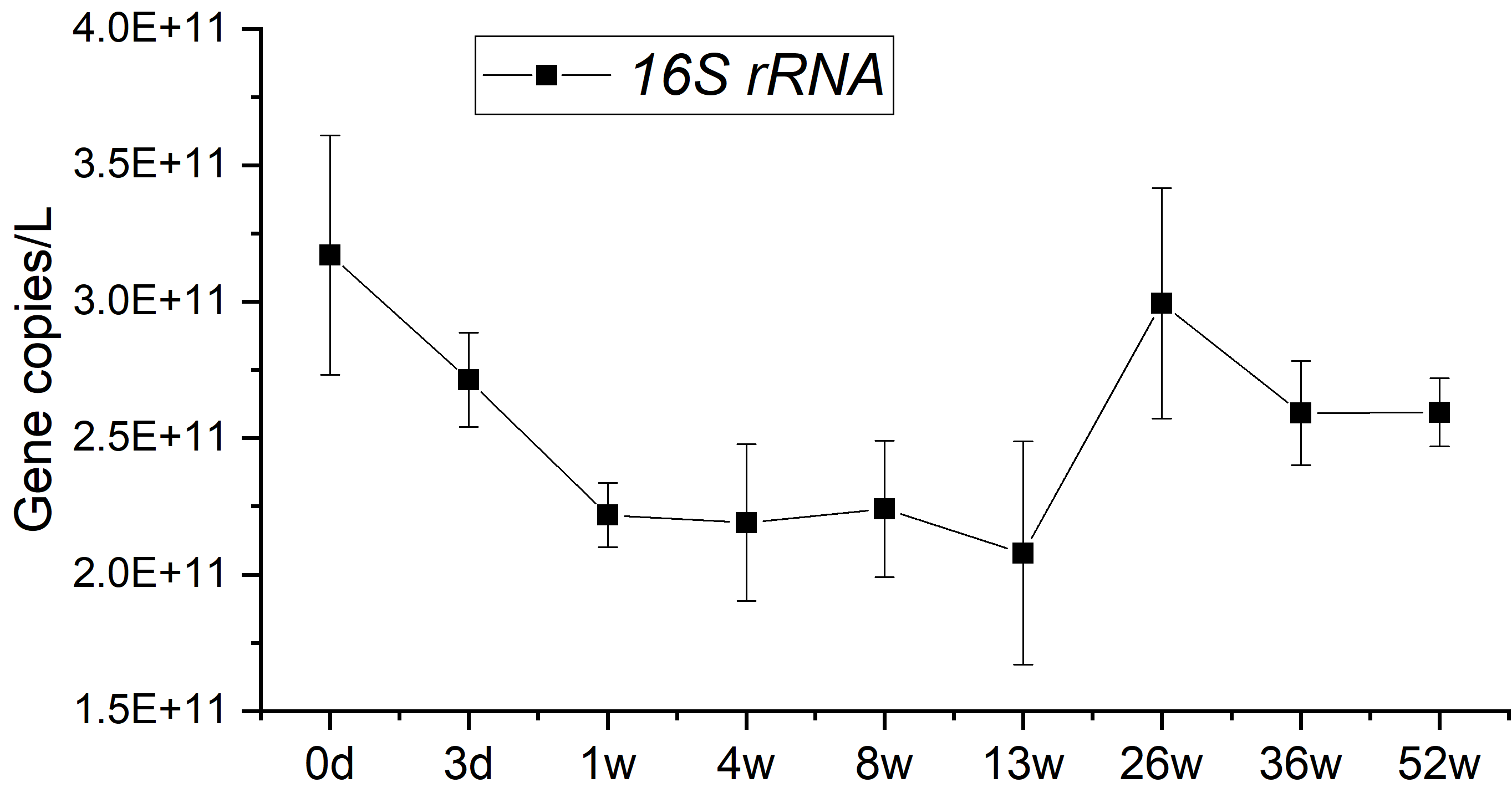  **15%** | 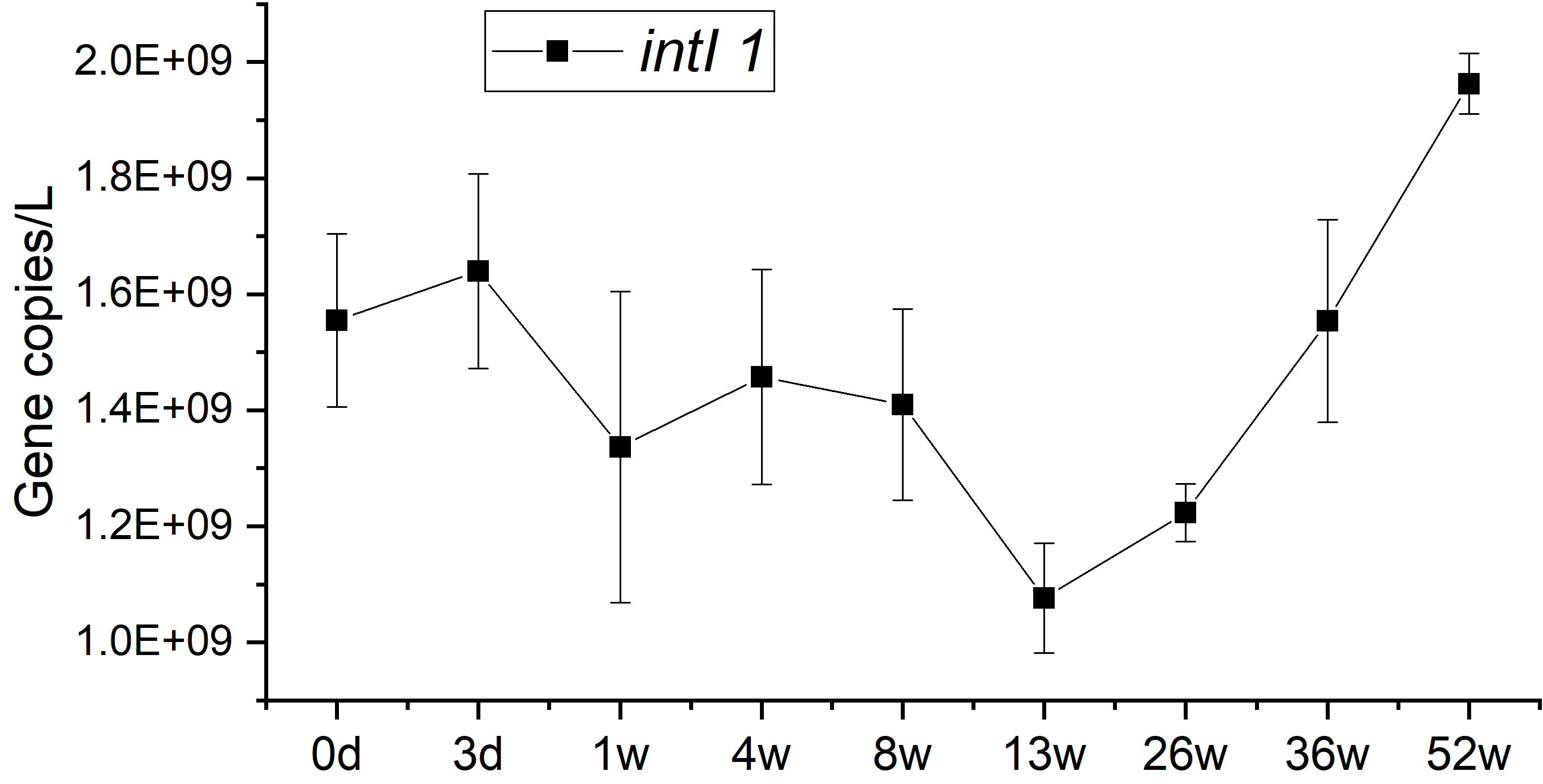  **17%** |
| --- | --- |
| 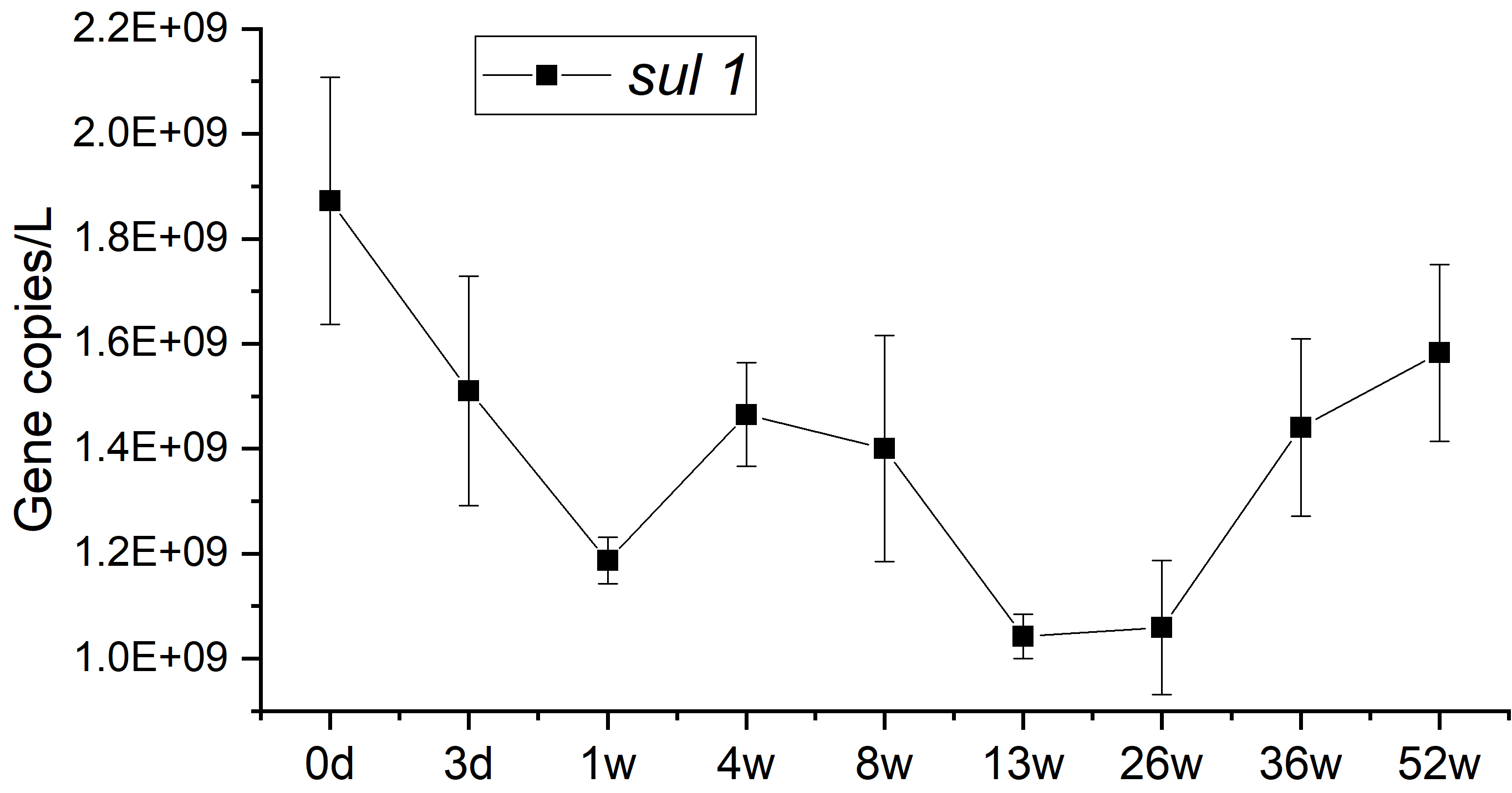  **19%** | 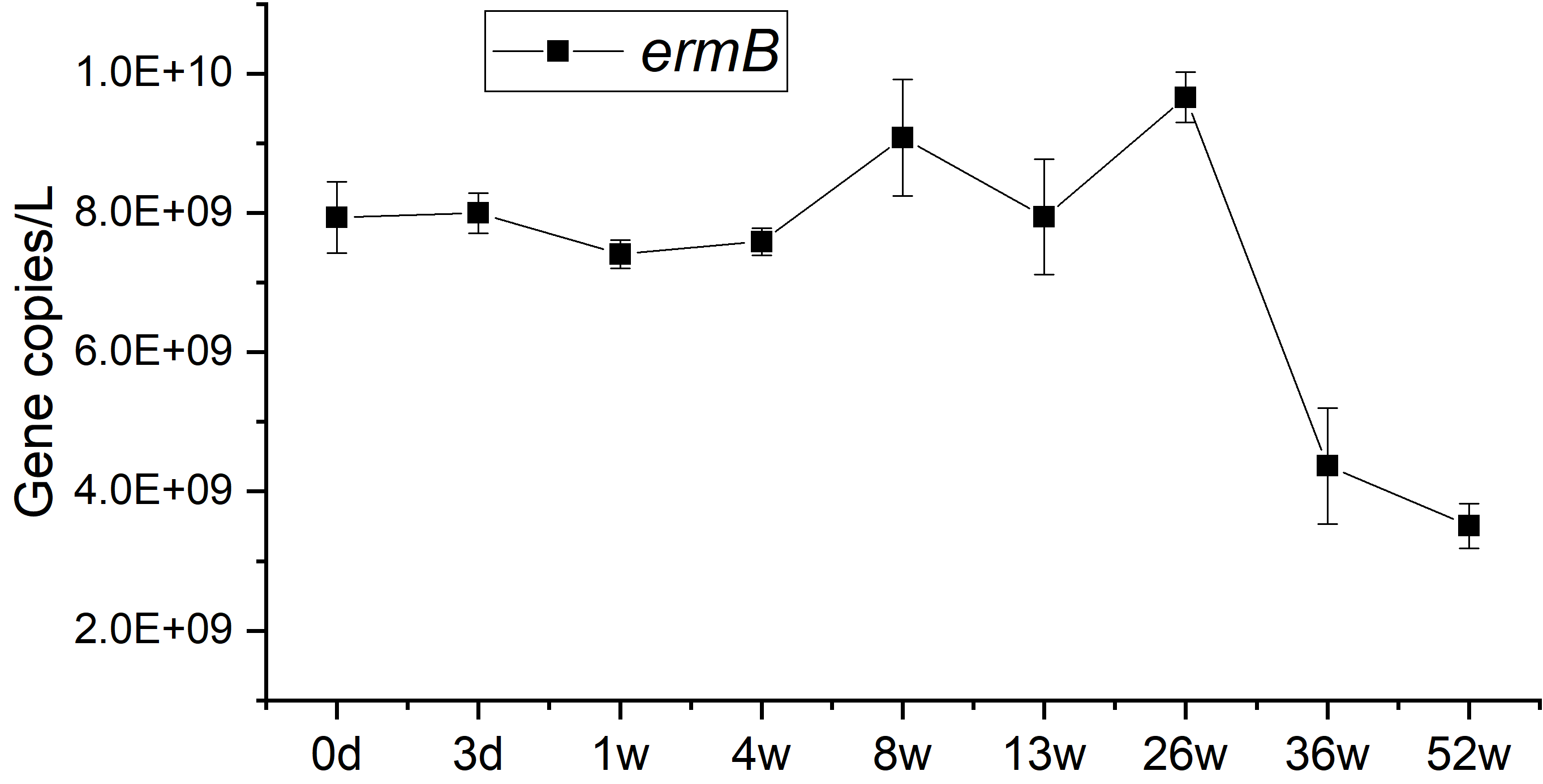 **28%** |
| 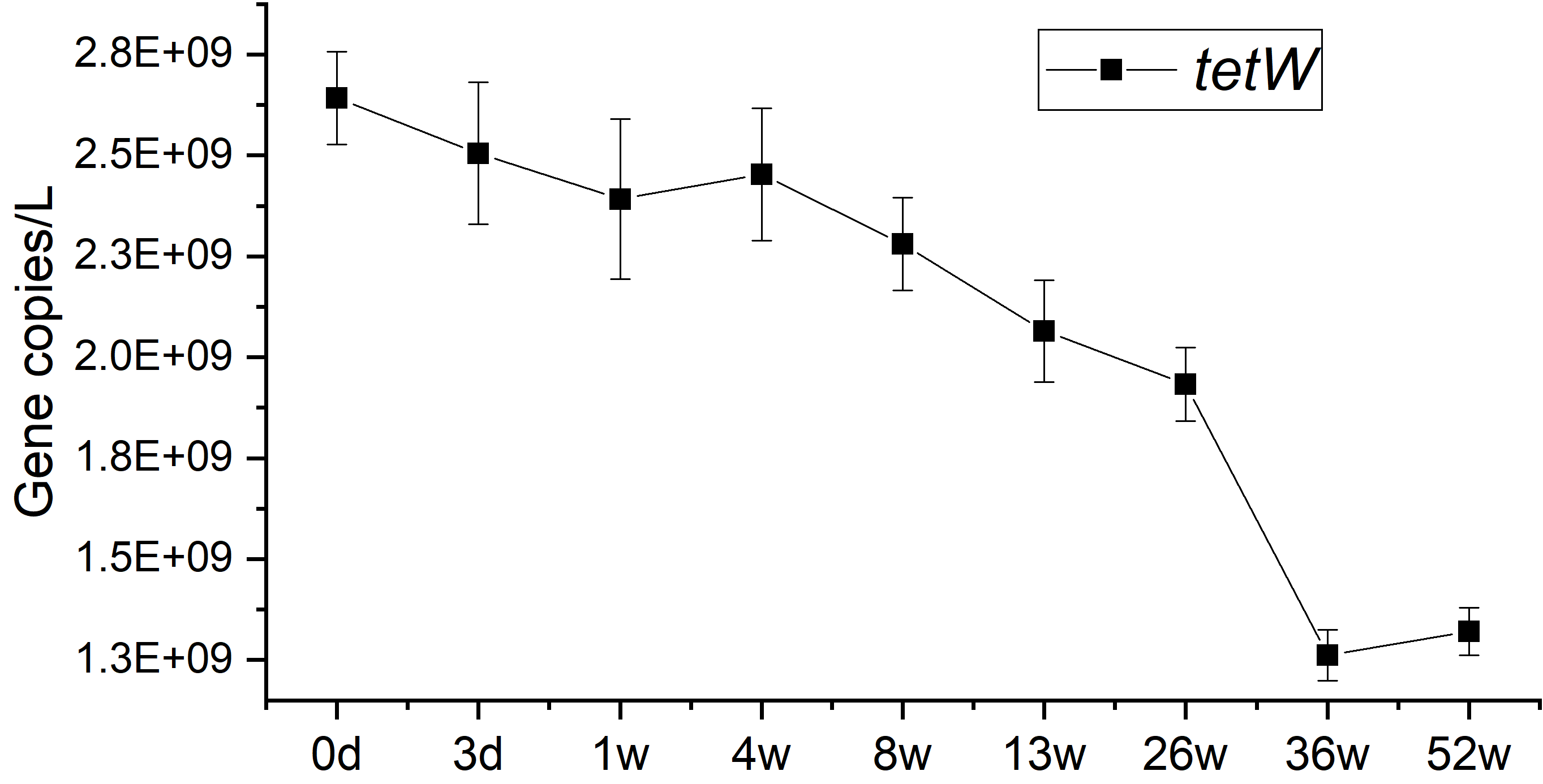  **24%** | 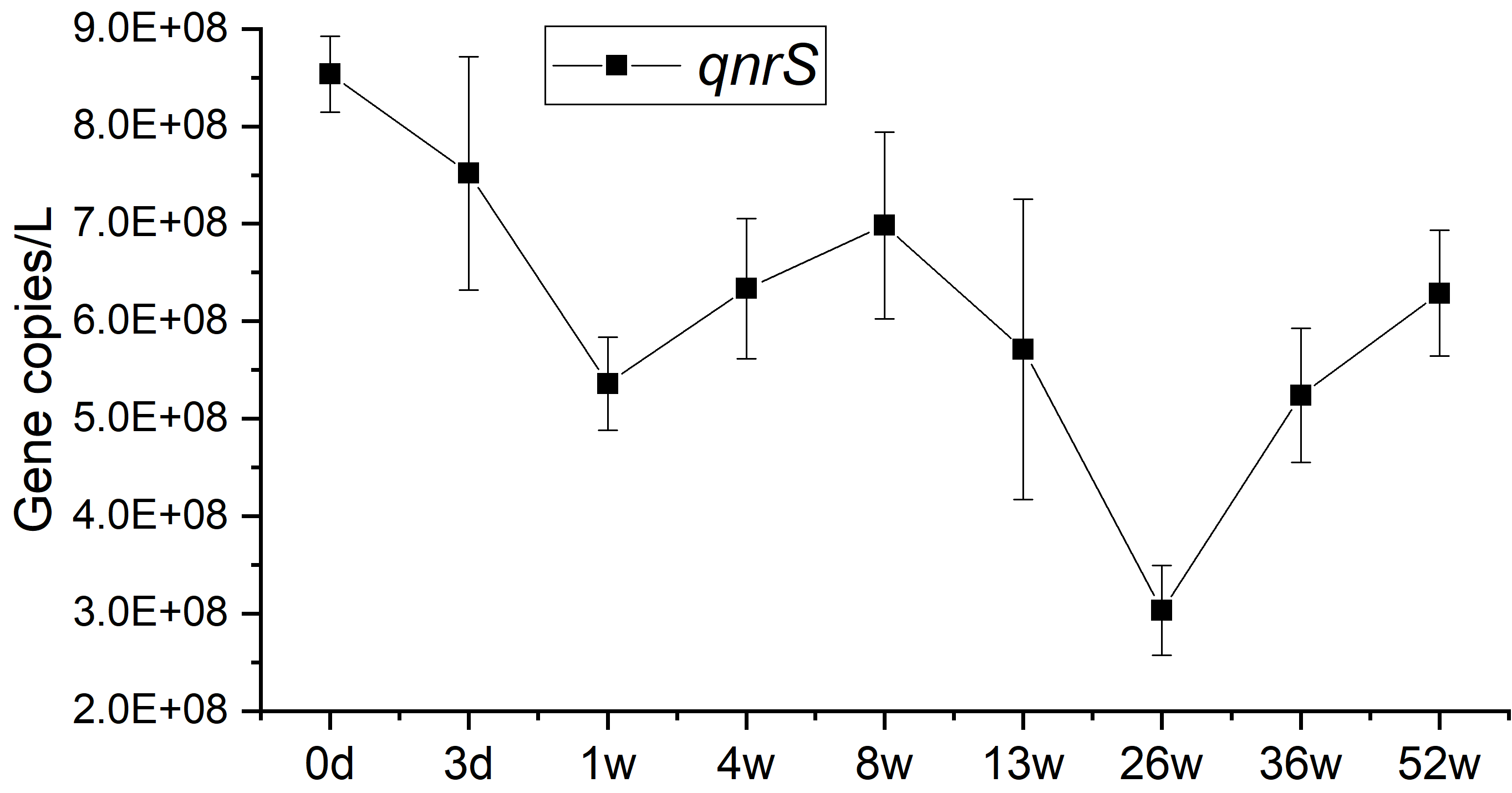  **26%** |
| 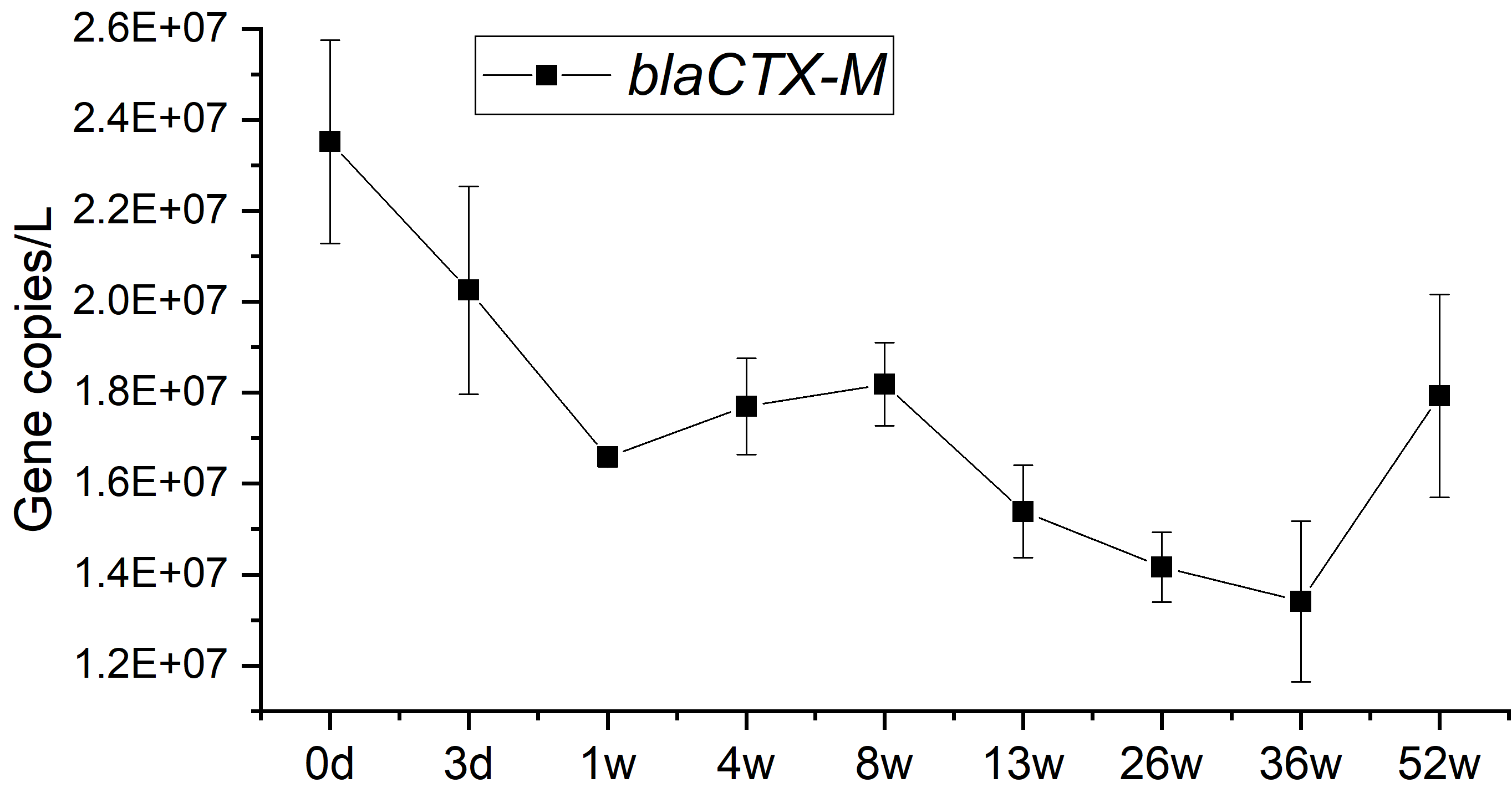  **18%** |  |
| Figure S2. Variation of target genes across 1-year freezing period. Results are provided as gene copies per litre and percentage variation. | |

| 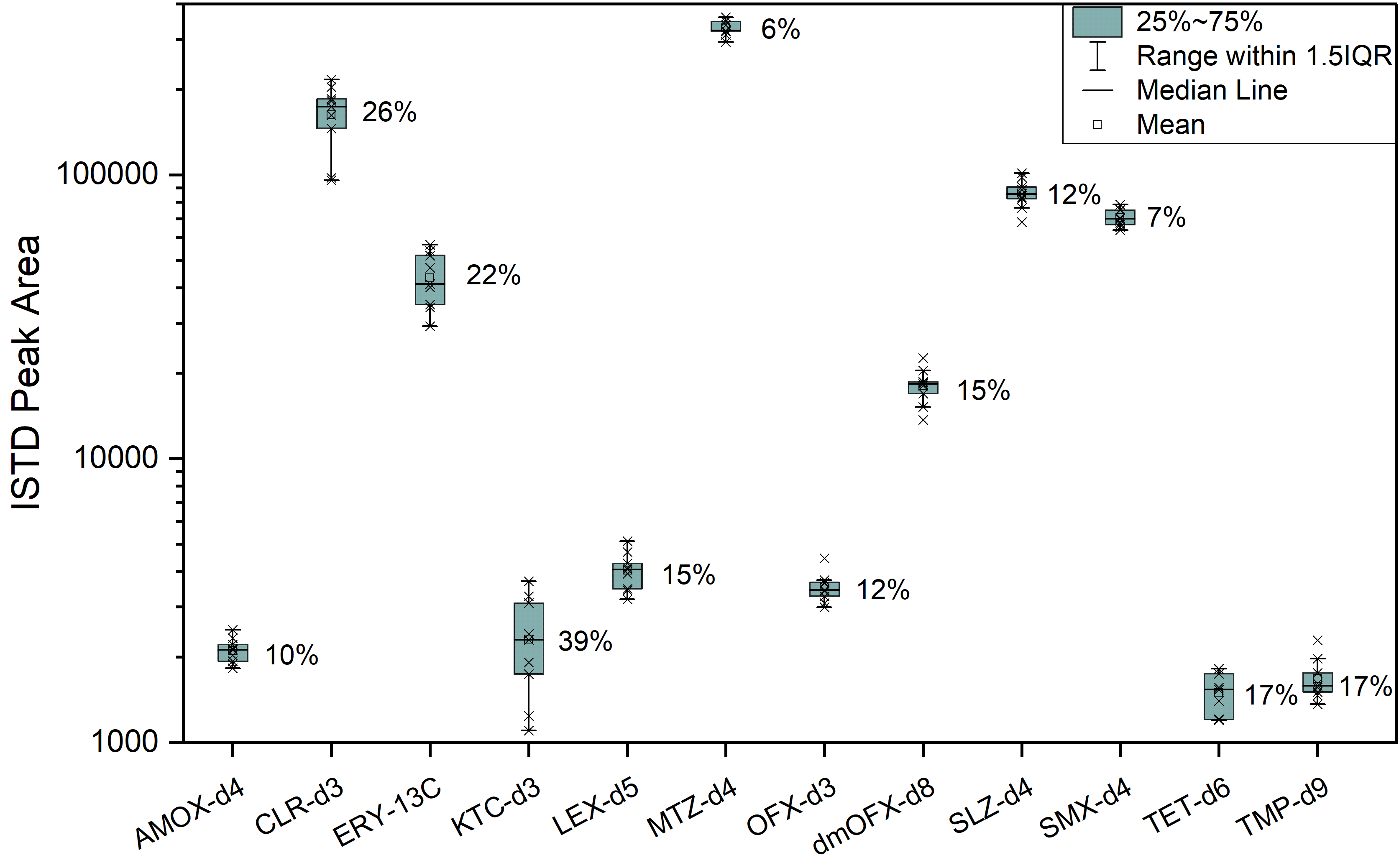 |
| --- |
| Figure S3. Box plot showing internal standard peak areas throughout the stability study. Percentage indicates the coefficient variance. |
